# Serum [‐2]proPSA as a Candidate Phenotypic Correlate of Molecular Alterations and Prognostic Marker in Metastatic Castration‐Resistant Prostate Cancer

**DOI:** 10.1155/proc/4207108

**Published:** 2026-10-28

**Authors:** Vincenza Conteduca, Marianna Garofoli, Giuseppina Bruno, Matteo Landriscina, Mariano Fiorenza, Carmela Polito, Rosa Sirica, Daniela Terracciano

**Affiliations:** ^1^ Unit of Medical Oncology and Biomolecular Therapy and CREATE Center for Research and Innovation Medicine Department of Medical and Surgical Sciences Policlinico Riuniti University of Foggia Foggia Italy unifg.it; ^2^ Department of Translational Medical Sciences University of Naples “Federico II” Naples 80131 Italy unina.it

## Abstract

**Background:**

Serum biomarkers that complement molecular profiling and provide prognostic information are of interest in metastatic castration‐resistant prostate cancer (mCRPC).

**Methods:**

We performed a single‐centre retrospective exploratory analysis of 31 patients with mCRPC receiving systemic therapy. Baseline serum total PSA, free PSA, [‐2]proPSA (p2PSA) and prostate health index (PHI) were evaluated together with tumour somatic molecular alterations and overall survival (OS). Baseline p2PSA was analysed both continuously after log2 transformation and using an exploratory 300 pg/mL threshold. On‐treatment p2PSA change was reanalysed as a time‐dependent covariate because follow‐up sampling occurred at variable times.

**Results:**

Median age was 75.1 years (range 60.2–83.2). Median follow‐up was 38.3 months (95% CI 22.5–42.8). Each doubling of baseline p2PSA was associated with shorter OS (HR 1.51, 95% CI 1.16–1.96; *p* = 0.0019). Patients with p2PSA > 300 pg/mL (*n* = 8; 6 deaths) had shorter OS than those with p2PSA ≤ 300 pg/mL (*n* = 23; 8 deaths; HR 4.61, 95% CI 1.46–14.56; log‐rank *p* = 0.0043). Total PSA, free PSA and PHI were also associated with OS, and adding p2PSA to total PSA did not significantly improve model fit (likelihood‐ratio *p* = 0.156). Baseline p2PSA was higher in patients with a pathogenic/likely pathogenic somatic alteration detected (*n* = 9) than in those with no pathogenic alteration detected (*n* = 22; Mann–Whitney *p* = 0.0348; Cliff’s delta 0.49), but this association attenuated after exclusion of extreme values. On‐treatment p2PSA change was not associated with OS after time‐dependent modelling (failure to achieve ≥ 50% decline: HR 1.44, 95% CI 0.42–4.90; *p* = 0.557).

**Conclusions:**

In this small hypothesis‐generating mCRPC cohort, higher baseline p2PSA was associated with poorer OS and with the presence of pathogenic/likely pathogenic somatic tumour alterations. These findings support prospective validation of p2PSA as an exploratory prognostic marker and candidate phenotypic correlate, rather than a surrogate for tumour genomics.

## 1. Introduction

Metastatic castration‐resistant prostate cancer (mCRPC) is characterized by substantial clinical and molecular heterogeneity and variable outcomes [1, 2]. Molecular profiling can identify biologically and therapeutically relevant alterations, but its routine implementation may be limited by tissue availability, cost, timing and access [3, 4]. Easily measurable serum biomarkers that complement molecular information, therefore, remain of clinical interest.

Among PSA molecular forms, [‐2]proPSA (p2PSA) is preferentially associated with malignant prostate epithelium and is incorporated into the prostate health index (PHI). p2PSA‐derived indices have been studied predominantly in diagnosis and risk stratification [5, 6]. More recently, PSA isoforms and their kinetics have been explored in mCRPC [7], raising the hypothesis that p2PSA may also carry information on tumour biology and prognosis in advanced disease.

The present exploratory study aimed to evaluate the association of baseline serum p2PSA with OS in mCRPC, compare its prognostic behaviour with total PSA, free PSA and PHI and explore the relationship between p2PSA and tumour somatic molecular alterations. We also reevaluated on‐treatment p2PSA change while explicitly accounting for variable sampling time and guarantee‐time bias.

## 2. Materials and Methods

### 2.1. Study Design and Population

This retrospective, single‐centre exploratory study analysed 31 evaluable patients with mCRPC treated at the Unit of Medical Oncology and Biomolecular Therapy, University of Foggia/Policlinico Riuniti. In the verified patient‐level dataset, treatment initiation used as time zero occurred between 25 October 2021 and 24 January 2025; the latest recorded follow‐up was 20 January 2026, which was used as the provisional database‐lock date for the present reanalysis. All patients had mCRPC at the treatment baseline, remained on androgen‐deprivation therapy and received systemic treatment according to standard clinical practice. Treatment line was first line in 16 patients, second line in 11 and third line in 4.

The study population consisted of 31 patients with documented mCRPC for whom baseline serum samples, clinical follow‐up data and tumour molecular testing were available. Baseline total PSA, free PSA, p2PSA and PHI were available for all patients, and all 31 patients also had an on‐treatment serum sample. Castration‐resistant status was defined clinically by disease progression despite castrate androgen levels and continuation of ADT, consistent with the approach used in prior mCRPC studies from the clinical group [1, 7]. ECOG performance status, treatment line, metastatic sites, disease burden, subsequent therapy and survival status were abstracted from the clinical database. Metastatic sites were nonmutually exclusive.

### 2.2. Serum Biomarker Measurements

Blood samples were processed according to the laboratory protocol used in prior p2PSA studies from our laboratory [6]. Serum was separated within 3 h of collection and stored at −80°C until analysis. Serum total PSA, free PSA and [‐2]proPSA (p2PSA) were measured using Beckman Coulter Access Hybritech immunoassays on an Access 2 Immunoassay System (Beckman Coulter, Brea, CA, USA). Serum was obtained from blood collected without anticoagulants and separated by centrifugation. According to the manufacturer’s instructions, specimens should be processed and refrigerated within 3 h of collection; samples analysed within 24 h may be stored at 2°C–8°C, whereas longer term storage requires freezing at −20°C or below and at −70°C for storage exceeding 5 months. Repeated freeze–thaw cycles have not been reported to significantly affect total PSA, free PSA or p2PSA measurements.

The Access Hybritech p2PSA assay has a limit of blank of 0.50 pg/mL and a limit of quantification of 3.23 pg/mL. The analytical measuring range extends to approximately 5000 pg/mL, corresponding to the highest calibrator concentration. Total assay imprecision is reported by the manufacturer to be < 20% between the LoQ and 10 pg/mL and ≤ 10% at concentrations ≥ 10 pg/mL; within‐run CVs in the manufacturer’s precision study ranged approximately from 2.2% to 4.9%, with total CVs from 2.9% to 6.1%.

Calibration was performed using the manufacturer’s multipoint calibration procedure, with calibrators spanning approximately 0–5000 pg/mL; an active calibration curve is required, and recalibration is recommended every 28 days. Quality‐control materials covering at least two analyte concentrations were used according to the manufacturer’s recommendations.

Percentage p2PSA change was calculated as [(follow‐up p2PSA − baseline p2PSA)/baseline p2PSA] × 100. Because sampling time was variable: median 93 days after baseline (IQR 54–155.5; range 14–309 days), on‐treatment p2PSA change was evaluated using a time‐dependent Cox model. A ≥ 50% decline was retained as an exploratory clinically interpretable threshold.

### 2.3. Tumour Molecular Testing

All 31 patients had tumour somatic molecular testing performed on tissue, and the timing of testing was available in the clinical database.

Only tumour somatic alterations were included in the molecular‐status analyses reported in this study. Germline testing was performed as part of the broader molecular work‐up but was not used to define molecular‐status groups and was not included in the present association or survival analyses.

For the main analyses, patients were classified according to somatic molecular status as “pathogenic/likely pathogenic alteration detected” or “no pathogenic alteration detected.” Detected alterations involved TP53 (*n* = 3), PTEN (*n* = 2), BRCA2 (*n* = 1), ATM (*n* = 1), CHEK2 (*n* = 1) and PALB2 (*n* = 1); no overlapping alteration categories were recorded in the supplied dataset.

For completeness, germline testing performed as part of the clinical molecular work‐up is described below, although germline findings were not included in the analyses reported here.

Germline DNA was extracted from peripheral blood lymphocytes using the MagCore HF16 automated system, while tumour DNA was isolated from FFPE primary prostate cancer tissues using the QIAamp DNA FFPE kit. Genomic libraries were prepared using targeted NGS panels comprising 38 genes for germline analysis and 20 genes for somatic analysis (SOPHiA GENETICS). Sequencing was performed on the Illumina MiSeq platform, with minimum coverage of 50*x* and 1000*x* for germline and somatic DNA, respectively. SNVs, indels and CNVs were analysed using the SOPHiA DDM platform, and variants were classified according to HGVS nomenclature. All sequencing and analyses for the primary cohort were conducted at the University Hospital of Foggia, Italy.

### 2.4. Outcome and Statistical Analysis

Overall survival (OS) was calculated from treatment initiation to death from any cause or last follow‐up. Survival distributions were estimated using the Kaplan–Meier method. For the exploratory analysis based on the 300 pg/mL p2PSA threshold, survival curves for patients with baseline p2PSA > 300 and ≤ 300 pg/mL were compared using the log‐rank test. Cox proportional‐hazards regression was used to estimate hazard ratios (HRs) and 95% confidence intervals (CIs). Baseline biomarkers were analysed as continuous log2‐transformed variables so that HRs represent the relative hazard associated with each biomarker doubling. The 300 pg/mL threshold was retained as an exploratory secondary analysis and is not presented as validated or prespecified. Given only 14 deaths, conventional multivariable modelling with multiple covariates was avoided. Exploratory two‐predictor Cox models were used only as sensitivity analyses. In parsimonious two‐predictor sensitivity models, baseline log2 p2PSA remained significantly associated with OS when p2PSA was entered separately with treatment line, ECOG performance status, treatment type or disease burden. Given the limited number of deaths, these models were considered sensitivity analyses rather than conventional multivariable adjustment. Incremental fit of p2PSA beyond total PSA was assessed by likelihood‐ratio testing. Harrell’s concordance index was reported descriptively. The proportional‐hazards assumption was assessed using Schoenfeld residuals; no evidence of violation was observed for baseline p2PSA (continuous log2 p2PSA, *p* = 0.771; dichotomized p2PSA, *p* = 0.488). Breslow handling of ties was used, and complete‐case analysis was applied; all primary biomarker and survival variables were complete.

Because on‐treatment sampling occurred at variable times, p2PSA change was modelled as a time‐dependent covariate, becoming available at the actual follow‐up sampling time. This approach avoids assigning future biomarker information at baseline. A fixed 1‐month landmark analysis was not performed because the second sample was not obtained at a uniform 1‐month landmark.

Baseline p2PSA distributions by molecular status were compared using a two‐sided Mann–Whitney U test. Effect size was quantified by Cliff’s delta with a bootstrap 95% CI. Because p2PSA was markedly right‐skewed, individual‐patient values are displayed on a logarithmic axis. Sensitivity analyses sequentially excluded the highest p2PSA observations. Gene‐specific analyses were descriptive only because of very small subgroup sizes. Two‐sided *p* < 0.05 was considered statistically significant. Analyses for the revision were performed using Python 3.13 with statsmodels 0.14.6 and SciPy. Reporting was revised with reference to STROBE and REMARK principles.

## 3. Results

### 3.1. Study Population

Thirty‐one patients were included. Median age at treatment baseline was 75.1 years (range 60.2–83.2), and 14 deaths occurred. Median follow‐up was 38.3 months (95% CI 22.5–42.8). ECOG performance status was 0 in 5 patients, 1 in 21 and 2 in 5. Disease burden was high volume in 22 (71%) and low volume in 9 (29%). Current mCRPC treatment was enzalutamide in 12, docetaxel in 11, apalutamide in 5 and abiraterone in 3 patients. Full clinical and laboratory characteristics are reported in Supporting Table S1. Supporting Figure S1 summarizes the study cohort, biomarker data availability, and timing of on‐treatment serum sampling in the 31 evaluable patients.

### 3.2. Baseline p2PSA and OS

Baseline p2PSA was markedly right‐skewed (median 133.45 pg/mL; IQR 27.12–286.44; range 1.11–16,220.26). Analysed continuously, each doubling of baseline p2PSA was associated with shorter OS (HR 1.51, 95% CI 1.16–1.96; *p* = 0.0019; Harrell C‐index 0.774). Using the exploratory threshold of 300 pg/mL, 8 patients had high p2PSA (> 300 pg/mL; 6 deaths) and 23 had p2PSA ≤ 300 pg/mL (8 deaths). High baseline p2PSA was associated with shorter OS (HR 4.61, 95% CI 1.46–14.56; *p* = 0.0091; log‐rank *p* = 0.0043; Figure 1A). Median OS was 19.3 months in the > 300 pg/mL group and 40.8 months in the ≤ 300 pg/mL group; CIs were wide, and upper bounds were not estimable in this small cohort.

**FIGURE 1:**
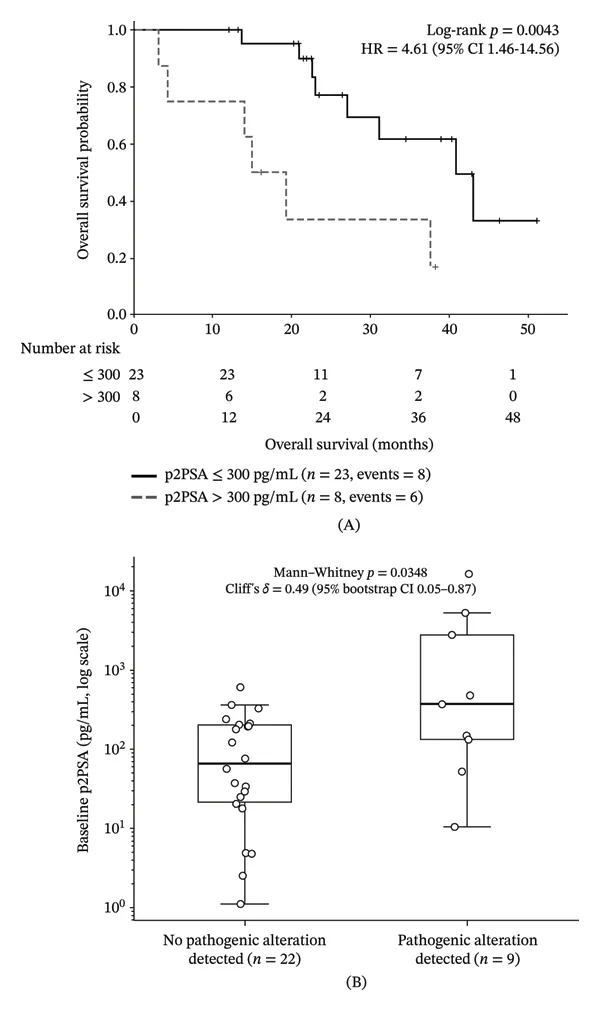
(A) Kaplan–Meier overall survival according to the exploratory baseline p2PSA threshold of 300 pg/mL. High p2PSA (> 300 pg/mL; *n* = 8, 6 deaths) was associated with shorter OS than p2PSA ≤ 300 pg/mL (*n* = 23, 8 deaths; Cox HR 4.61, 95% CI 1.46–14.56; log‐rank *p* = 0.0043). (B) Individual baseline p2PSA values according to tumour molecular status, shown on a logarithmic scale. Patients with a pathogenic/likely pathogenic somatic alteration detected (*n* = 9) had higher baseline p2PSA than patients with no pathogenic alteration detected (*n* = 22; Mann–Whitney *p* = 0.0348; cliff’s delta 0.49, bootstrap 95% CI 0.05–0.87).

### 3.3. Comparison With Other PSA‐Related Biomarkers

Total PSA, free PSA and PHI were also associated with OS when analysed per biomarker doubling: total PSA HR 1.34 (95% CI 1.11–1.63; *p* = 0.0030), free PSA HR 1.44 (95% CI 1.14–1.81; *p* = 0.0022) and PHI HR 1.62 (95% CI 1.18–2.22; *p* = 0.0028). Corresponding Harrell C‐indices were 0.749, 0.766 and 0.774, respectively. Percentage p2PSA was not associated with OS (HR 1.04 per doubling, 95% CI 0.48–2.25; *p* = 0.928). In an exploratory model containing both total PSA and p2PSA, adding p2PSA did not significantly improve fit over total PSA alone (likelihood‐ratio *p* = 0.156).

### 3.4. Exploratory Clinical Sensitivity Analyses

In univariable exploratory analyses, higher treatment line was associated with shorter OS (HR 3.59 per line, 95% CI 1.47–8.75; *p* = 0.0050), and high disease burden was associated with shorter OS (HR 8.08, 95% CI 1.05–62.18; *p* = 0.0446). ECOG performance status did not reach statistical significance (HR 2.19 per category, 95% CI 0.86–5.55; *p* = 0.099). Treatment with docetaxel versus nondocetaxel therapy was not associated with OS (*p* = 0.742). Because the event count was small, these analyses are descriptive and no conventional multivariable model is claimed. In parsimonious two‐predictor sensitivity models, baseline log2 p2PSA remained significantly associated with OS when p2PSA was entered separately with treatment line, ECOG performance status, treatment type or disease burden. Full coefficients, HRs, 95% CIs and values for these models are provided in Supporting Table S2. Given the limited number of deaths, these models were considered sensitivity analyses rather than conventional multivariable adjustment.

All 31 patients had an on‐treatment sample, but timing was heterogeneous (median 93 days; IQR 54–155.5; range 14–309). Eleven patients achieved a ≥ 50% p2PSA decline, and 20 did not. A naive baseline classification by future response was not associated with OS (HR 1.20, 95% CI 0.36–4.00; *p* = 0.765). More importantly, after modelling response as a time‐dependent covariate at the actual sampling time, failure to achieve a ≥ 50% decline was not associated with OS (HR 1.44, 95% CI 0.42–4.90; *p* = 0.557). Continuous log2 on‐treatment/baseline p2PSA change was likewise not associated with OS (HR 1.06, 95% CI 0.81–1.38; *p* = 0.672).

### 3.5. p2PSA and Tumour Molecular Alterations

Nine of 31 patients (29.0%) had at least one pathogenic/likely pathogenic somatic tumour alteration detected, whereas 22 (71.0%) had no pathogenic alteration detected. Baseline p2PSA was higher in patients with a pathogenic alteration detected (median 374.56 pg/mL; IQR 133.45–2787.30; range 10.44–16,220.26) than that in patients with no pathogenic alteration detected (median 66.57 pg/mL; IQR 21.45–202.75; range 1.11–612.27; Mann–Whitney *p* = 0.0348; Cliff’s delta 0.49, bootstrap 95% CI 0.05–0.87; Figure 1B).

The molecular association was sensitive to extreme observations. After removal of the single highest p2PSA value, *p* = 0.078; after sequential exclusion of the two and three highest values, *p* = 0.181 and *p* = 0.395, respectively. Therefore, this finding should be regarded as exploratory. Gene‐specific sample sizes were extremely small (TP53 *n* = 3, PTEN *n* = 2, BRCA2 *n* = 1, ATM *n* = 1, CHEK2 *n* = 1 and PALB2 *n* = 1), precluding inferential comparisons or claims of a gene‐specific gradient (Supporting Figure S2). Binary molecular status itself was not significantly associated with OS (HR 1.74, 95% CI 0.51–5.89; *p* = 0.377).

## 4. Discussion

The principal finding of this exploratory study is that baseline serum p2PSA is associated with OS in mCRPC. Importantly, the association was observed when p2PSA was treated as a continuous log‐transformed variable, reducing reliance on a data‐dependent dichotomous threshold. The 300 pg/mL grouping remains useful only as an exploratory visualization and should not be interpreted as a validated clinical cutoff.

In the context of related PSA measures, total PSA, free PSA and PHI were all associated with OS, and PHI showed a concordance index similar to p2PSA. Furthermore, p2PSA did not demonstrate significant incremental model fit beyond total PSA in this small cohort. These findings argue against interpreting p2PSA as a uniquely independent measure of tumour biology and instead support its evaluation as one component of a broader biomarker framework.

Serum p2PSA should also be interpreted within the broader landscape of minimally invasive biomarkers developed for prostate cancer risk stratification. Matuszczak et al. reviewed clinically available blood‐ and urine‐based assays, including PHI, 4Kscore, PCA3, SelectMDx, ExoDx, MyProstateScore and Stockholm3, highlighting both their potential to complement conventional PSA testing and the need for context‐specific validation and demonstration of clinical utility [8]. In this framework, the present results raise the hypothesis that p2PSA may have relevance beyond early detection and may be associated with an adverse molecular tumour phenotype. Patients with a pathogenic/likely pathogenic somatic alteration detected had higher baseline p2PSA values than patients with no pathogenic alteration detected.

However, this association weakened after exclusion of the highest p2PSA observations, indicating that the finding was influenced by extreme values and should, therefore, be considered exploratory. The alterations identified in our cohort involved TP53, PTEN, BRCA2, ATM, CHEK2 and PALB2. Several of these genes are clinically relevant in advanced prostate cancer: Alterations involving TP53 and PTEN have been associated with aggressive disease and adverse outcomes, while BRCA2 alterations have both prognostic and established therapeutic relevance in the mCRPC setting [9, 10]. Nevertheless, each individual molecular subgroup in our cohort was represented by very few patients, precluding any gene‐specific prognostic inference. Rather than suggesting a direct relationship with a specific genomic alteration, our findings raise the hypothesis that higher circulating p2PSA may reflect a more adverse molecular tumour phenotype. This hypothesis requires confirmation in larger, prospectively characterized and molecularly stratified cohorts. Modelling on‐treatment p2PSA change as a time‐dependent covariate, thereby addressing guarantee‐time bias, did not show an association with OS. However, the variable timing of follow‐up sampling and the limited number of events may have reduced the ability to detect a true association, and the prognostic relevance of p2PSA kinetics should, therefore, be reassessed in larger prospective cohorts with standardized sampling time points.

This study has substantial limitations. It is retrospective and single‐center, with only 31 patients and 14 deaths, creating risk of selection bias and unstable estimates. Treatment type, treatment line, prior therapy and disease burden were heterogeneous. A formal screening log was unavailable. The 300 pg/mL threshold was exploratory rather than externally validated. Multiple analyses were performed without external validation. The p2PSA distribution contained influential extreme values, particularly among molecularly altered cases. The variable timing of follow‐up blood collection precluded a fixed 1‐month landmark analysis.

In conclusion, baseline serum p2PSA was associated with OS in this small exploratory mCRPC cohort and was higher among patients with a pathogenic/likely pathogenic somatic alteration detected. On‐treatment p2PSA change was not prognostic after accounting for its timing. These findings are preliminary and hypothesis‐generating and warrant validation in larger, prospectively collected cohorts with standardized sampling and comprehensive molecular profiling.

## Author Contributions

Daniela Terracciano and Vincenza Conteduca conceived and finalized the manuscript. Laboratory analyses were conducted by Daniela Terracciano, Mariano Fiorenza, Carmela Polito and Rosa Sirica, while clinical assessment was performed by Vincenza Conteduca, Marianna Garofoli, Giuseppina Bruno and Matteo Landriscina. Daniela Terracciano and Vincenza Conteduca supervised the study.

## Funding

This study was supported by the AIRC Investigator Grant 2023: 29370; Ministry of University and Research (PRIN—Progetti di Ricerca di Rilevante Interesse Nazionale—Università degli Studi di Foggia—CUP D53D23014290006); Italian Ministry of Health under PNRR‐M6/C2 Project PNRR‐MR1‐2023‐12377571 and European Union‐NextGenerationEU through the Italian Ministry of University and Research under PNRR‐M4C2‐I1.3 Project PE_00000019 “HEAL ITALIA,” CUP UNIFG D73C22001230006.

## Disclosure

The funding sources had no role in study design, collection, analysis or interpretation of data, writing of the report or the decision to submit the report for publication. All authors reviewed and approved the final manuscript.

## Ethics Statement

Written informed consent was obtained from all participants before enrolment. The study was conducted in accordance with the principles of the Declaration of Helsinki and was approved by the Ethics Committee of the University of Naples Federico II (Project identification code 118/20).

## Conflicts of Interest

Vincenza Conteduca has served as a consultant/advisory board member for Johnson & Johnson, Astellas, Merck, AstraZeneca, Amgen, EISAI, Recordati, Novartis, Ipsen and Bayer and has received speaker honoraria or travel support from Johnson & Johnson, Astellas, Janssen, Ipsen, Bayer, Gilead, Novartis, Accord and Bristol Myers Squibb. Daniela Terracciano reports receiving honoraria for scientific lectures, educational grants and advisory board activities from Fujirebio, Novartis, Siemens and Roche, outside the submitted work.

## Supporting Information

Additional supporting information can be found online in the Supporting Information section.

## Supporting information


**Supporting Information** Supporting Table S1. Clinical, pathological, laboratory, treatment and molecular characteristics of the study population. Supporting Table S2. Complete Cox regression output and exploratory model‐comparison results. Supporting Figure S1. Flow of the retrospective biomarker dataset and data availability. Supporting Figure S2. Descriptive gene‐specific distribution of baseline p2PSA.

## Data Availability

The data supporting the findings of this study are available from the corresponding author upon reasonable request, subject to institutional and ethical restrictions.

## References

[1] Bruno G. , Natalicchio M. I. , Garofoli M. et al., Real-World Outcomes and Molecular Profiling for Patients With Metastatic Castration-Resistant Prostate Cancer With Lung Metastases: A Long-Term Multicenter Experience, European Urology Oncology. (2025) 8, no. 6, 1513–1523, 10.1016/j.euo.2025.05.002. 40461348

[2] Singhal U. , Jiang R. , Proudfoot J. A. et al., Characterizing Population-Wide Genomic Risk Distribution for Development of a Novel Clinical-Genomic Risk System for Prognostication in Patients With Clinically Localized Prostate Cancer, Prostate Cancer and Prostatic Diseases. (2026) 29, no. 3, 570–577, 10.1038/s41391-025-01062-8. PMC13472942 41366130

[3] Cucchiara V. , Cooperberg M. R. , Dall’Era M. et al., Genomic Markers in Prostate Cancer Decision Making, European Urology. (2018) 73, no. 4, 572–582, 10.1016/j.eururo.2017.10.036. 29129398

[4] Youssef J. , Yehya A. , Salhab Z. et al., Liquid Biopsy in Genitourinary Cancers: Diagnostic and Prognostic Implications, World Journal of Clinical Oncology. (2025) 16, no. 12, 10.5306/wjco.v16.i12.113578. PMC12754093 41480180

[5] Catalona W. J. , Partin A. W. , Sanda M. G. et al., A Multicenter Study of [-2]pro-Prostate Specific Antigen Combined With Prostate Specific Antigen and Free Prostate Specific Antigen for Prostate Cancer Detection in the 2.0 to 10.0 ng/mL Prostate Specific Antigen Range, Journal d’Urologie. (2011) 185, no. 5, 1650–1655, 10.1016/j.juro.2010.12.032. PMC3140702 21419439

[6] Ferro M. , Crocetto F. , La Civita E. et al., Serum (-2)proPSA/freePSA Ratio, (-2)proPSA/freePSA Density, Prostate Health Index, and Prostate Health Index Density as Clues to Reveal Postoperative Clinically Significant Prostate Cancer in Men With Prostate-Specific Antigen 2–10 ng/mL, The Prostate. (2024) 84, no. 12, 1157–1164, 10.1002/pros.24752. 38798011

[7] Fiala O. , Hošek P. , Korunková H. et al., Prognostic Role of Prostate-Specific Antigen Isoforms and Their Early Kinetics in Patients With Metastatic Castration-Resistant Prostate Cancer Receiving New Generation Androgen Receptor Targeted Agents, In Vivo. (2025) 39, no. 2, 859–869, 10.21873/invivo.13889. PMC11884487 40010964

[8] Matuszczak M. , Schalken J. A. , and Salagierski M. , Prostate Cancer Liquid Biopsy Biomarkers’ Clinical Utility in Diagnosis and Prognosis, Cancers (Basel). (2021) 13, no. 13, 10.3390/cancers13133373. PMC8268859 34282798

[9] Conteduca V. , Mosca A. , Brighi N. , de Giorgi U. , and Rescigno P. , New Prognostic Biomarkers in Metastatic Castration-Resistant Prostate Cancer, Cells. (2021) 10, no. 1, 10.3390/cells10010193. PMC7835961 33478015

[10] Fizazi K. , Attard G. , Azad A. A. et al., ESMO Guidelines Committee. Advanced and Metastatic Prostate Cancer: ESMO Clinical Practice Guideline for Diagnosis, Treatment and Follow-Up, Annals of Oncology. (2026) 37, no. 5, 590–607, 10.1016/j.annonc.2026.01.015. 41679466

