# Supplementary material for: Serum [‐2]proPSA as a Candidate Phenotypic Correlate of Molecular Alterations and Prognostic Marker in Metastatic Castration‐Resistant Prostate Cancer
Source: Prostate Cancer. 2026 Oct 28;2026:4207108. doi: 10.1155/proc/4207108 (PMC13717232; doi:10.1155/proc/4207108)
Supplement: Supplementary file 1 [file PROC-2026-4207108-s001.zip › Supplementary_Tables_S1_S2_v2.docx]

# Supplementary Table S1. Characteristics of the study population

| **Characteristic** | **Total (n=31)** |
| --- | --- |
| Patients analysed, n | 31 |
| Deaths, n (%) | 14 (45.2) |
| Median follow-up, reverse Kaplan–Meier, months (95% CI) | 38.3 (22.5–42.8) |
| Age at baseline, years, median (range) | 75.1 (60.2–83.2) |
| Gleason score <8, n (%) | 11 (35.5) |
| Gleason score ≥8, n (%) | 20 (64.5) |
| Prior prostatectomy: yes/no, n (%) | 14 (45.2) / 17 (54.8) |
| Prior radical radiotherapy: yes/no, n (%) | 6 (19.4) / 25 (80.6) |
| De novo/synchronous metastatic disease, n (%) | 12 (38.7) |
| Lymph-node metastases, n (%) | 19 (61.3) |
| Bone metastases, n (%) | 23 (74.2) |
| Visceral metastases, n (%) | 4 (12.9) |
| ECOG 0 / 1 / 2, n | 5 / 21 / 5 |
| Disease burden low / high, n (%) | 9 (29.0) / 22 (71.0) |
| mCRPC treatment: enzalutamide / docetaxel / apalutamide / abiraterone, n | 12 / 11 / 5 / 3 |
| Treatment line 1 / 2 / 3, n | 16 / 11 / 4 |
| Continuation of ADT | 31/31 (100%) |
| Baseline total PSA, ng/mL, median (IQR; range) | 9.93 (1.13–22.77; 0.047–4490.05) |
| Baseline free PSA, ng/mL, median (IQR; range) | 2.18 (0.48–5.44; 0.04–661.34) |
| Baseline p2PSA, pg/mL, median (IQR; range) | 133.45 (27.12–286.44; 1.11–16,220.26) |
| Baseline PHI, median (IQR; range) | 161.54 (57.52–226.55; 5.66–4092.50) |
| On-treatment p2PSA, pg/mL, median (IQR; range) | 42.26 (8.72–250.17; 1.00–25,679.05) |
| Baseline-to-follow-up sampling interval, days, median (IQR; range) | 93 (54–155.5; 14–309) |
| Achieved ≥50% p2PSA decline, n (%) | 11 (35.5) |
| Failed to achieve ≥50% p2PSA decline, n (%) | 20 (64.5) |
| Tumour somatic molecular testing available | 31/31 (100%); tissue-based |
| Pathogenic/likely pathogenic somatic alteration detected, n (%) | 9 (29.0) |
| No pathogenic alteration detected, n (%) | 22 (71.0) |
| Alteration categories, n | TP53 3; PTEN 2; BRCA2 1; ATM 1; CHEK2 1; PALB2 1 |
| PFS, months, median (IQR; range) | 11.8 (6.48–19.62; 1.4–37.77) |
| Observed OS, months, median (IQR; range) | 22.63 (17.72–37.92; 3.1–51.1) |

Footnotes: metastatic sites are non-mutually exclusive. ADT, androgen-deprivation therapy; ECOG, Eastern Cooperative Oncology Group; mCRPC, metastatic castration-resistant prostate cancer; PHI, prostate health index; PFS, progression-free survival; p2PSA, [-2]proPSA; PSA, prostate-specific antigen.

# Supplementary Table S2. Cox regression and exploratory prognostic analyses

| **Predictor** | **β** | **SE** | **Wald z** | **HR** | **95% CI** | **p** |
| --- | --- | --- | --- | --- | --- | --- |
| Baseline p2PSA >300 vs ≤300 pg/mL | 1.529 | 0.586 | 2.608 | 4.61 | 1.46–14.56 | 0.0091 |
| Baseline log2 p2PSA (per doubling) | 0.413 | 0.133 | 3.103 | 1.51 | 1.16–1.96 | 0.0019 |
| Baseline log2 total PSA (per doubling) | 0.294 | 0.099 | 2.968 | 1.34 | 1.11–1.63 | 0.0030 |
| Baseline log2 free PSA (per doubling) | 0.363 | 0.119 | 3.056 | 1.44 | 1.14–1.81 | 0.0022 |
| Baseline log2 PHI (per doubling) | 0.482 | 0.162 | 2.985 | 1.62 | 1.18–2.22 | 0.0028 |
| Baseline log2 %p2PSA (per doubling) | 0.036 | 0.396 | 0.090 | 1.04 | 0.48–2.25 | 0.9280 |
| Failure to achieve ≥50% decline (naive baseline coding) | 0.183 | 0.614 | 0.299 | 1.20 | 0.36–4.00 | 0.7653 |
| Failure to achieve ≥50% decline (time-dependent) | 0.367 | 0.624 | 0.588 | 1.44 | 0.42–4.90 | 0.5567 |
| Continuous log2 on-treatment/baseline p2PSA (time-dependent) | 0.058 | 0.136 | 0.423 | 1.06 | 0.81–1.38 | 0.6725 |
| Pathogenic alteration detected vs no pathogenic alteration detected | 0.551 | 0.624 | 0.884 | 1.74 | 0.51–5.89 | 0.3769 |
| High vs low disease burden | 2.090 | 1.041 | 2.008 | 8.08 | 1.05–62.18 | 0.0446 |
| Treatment line (per line) | 1.277 | 0.455 | 2.806 | 3.59 | 1.47–8.75 | 0.0050 |
| ECOG (per category) | 0.784 | 0.475 | 1.651 | 2.19 | 0.86–5.55 | 0.0987 |
| Docetaxel vs non-docetaxel | 0.190 | 0.576 | 0.330 | 1.21 | 0.39–3.74 | 0.7415 |
| Gleason ≥8 vs <8 | 0.585 | 0.659 | 0.887 | 1.80 | 0.49–6.54 | 0.3748 |

All Cox models use Breslow handling of ties. Univariable models are shown unless explicitly identified as time-dependent. Fourteen deaths occurred. Proportional-hazards assessment using Schoenfeld residuals showed no evidence of violation for baseline log2 p2PSA (p=0.771) or the >300 pg/mL indicator (p=0.488). Harrell C-indices: log2 p2PSA 0.774; log2 total PSA 0.749; log2 free PSA 0.766; log2 PHI 0.774. Adding log2 p2PSA to log2 total PSA did not significantly improve model fit (likelihood-ratio p=0.156).

**Exploratory two-predictor Cox sensitivity models**

| **Model** | **Predictor** | **HR** | **95% CI** | **p** |
| --- | --- | --- | --- | --- |
| Model 1 | Baseline log2 p2PSA (per doubling) | 1.49 | 1.13–1.96 | 0.0050 |
|  | Treatment line (per line) | 3.04 | 1.18–7.88 | 0.0217 |
| Model 2 | Baseline log2 p2PSA (per doubling) | 1.53 | 1.18–1.98 | 0.0011 |
|  | ECOG performance status (per category) | 2.49 | 0.99–6.28 | 0.0525 |
| Model 3 | Baseline log2 p2PSA (per doubling) | 1.53 | 1.18–1.97 | 0.0012 |
|  | Docetaxel vs non-docetaxel | 0.66 | 0.21–2.10 | 0.4866 |
| Model 4 | Baseline log2 p2PSA (per doubling) | 1.46 | 1.05–2.04 | 0.0263 |
|  | High vs low disease burden | 1.53 | 0.12–18.93 | 0.7385 |

**Notes.** All models included baseline log2-transformed p2PSA plus one clinical covariate. Cox models used Breslow handling of ties. Fourteen deaths occurred among 31 patients. Because of the limited number of events, these two-predictor models were used as parsimonious sensitivity analyses and should not be interpreted as fully adjusted multivariable models.

**Abbreviations:** CI, confidence interval; ECOG, Eastern Cooperative Oncology Group; HR, hazard ratio; p2PSA, [-2]proPSA.

## Sensitivity analysis for molecular-status comparison

| **Analysis** | **Pathogenic-alteration-detected group** | **Mann–Whitney p** |
| --- | --- | --- |
| Primary analysis | 9 | 0.0348 |
| Exclude highest p2PSA value | 8 | 0.0784 |
| Exclude two highest p2PSA values | 7 | 0.1811 |
| Exclude three highest p2PSA values | 6 | 0.3954 |

Primary effect size: Cliff’s delta 0.49; bootstrap 95% CI 0.05–0.87. These sensitivity analyses demonstrate that the molecular association is influenced by extreme observations and should be interpreted as exploratory.
